# Patient Attitudes Toward Ambient Voice Technology: Preimplementation Patient Survey in an Academic Medical Center

**DOI:** 10.2196/77901

**Published:** 2025-11-27

**Authors:** Gary Leiserowitz, Jeff Mansfield, Scott MacDonald, Melissa Jost

**Affiliations:** 1 University of California Davis Medical Center Sacramento, CA United States

**Keywords:** artificial intelligence, AI, AI scribe, scribe program, ambient voice technology, preimplementation patient survey, patient perceptions and attitudes, electronic health records, EHR, health information privacy

## Abstract

**Background:**

Many institutions are in various stages of deploying an artificial intelligence (AI) scribe system for clinic electronic health record (EHR) documentation. In anticipation of the University of California, Davis Health’s deployment of an AI scribe program, we surveyed current patients about their perceptions of this technology to inform a patient-centered implementation.

**Objective:**

We assessed patient perceptions about current clinician EHR documentation practices before implementation of the AI scribe program, and preconceptions regarding the AI scribe’s introduction.

**Methods:**

We conducted a descriptive preimplementation survey as a quality improvement study. A convenience sample of 9171 patients (aged ≥18 years) who had a clinic visit within the previous year, was recruited via an email postvisit survey. Patient-identified demographics (age, gender, and race and ethnicity) were collected. The survey included rating scales on questions related to the patient perception of the AI scribe program, plus open-ended comments. Data were collated to analyze patient perceptions of including AI Scribe technology in a clinician visit.

**Results:**

In total, 1893 patients completed the survey (20% response rate), with partial responses from another 549. Sixty-three percent (n=1205) of the respondents were female, and most were 51 years and older (87%, n=1649). Most patients identified themselves as White (69%, n=1312), multirace (8%, n=154), Latinx (7%, n=130), and Black (2%, n=42). The respondents were not representative of the overall clinic populations and skewed more toward being female, ages 50 years and older, and White in comparison. Patients reacted to the current EHR documentation system, with 71% (n=1349) feeling heard or sometimes heard, but 23% (n=416) expressed frustrations that their physician focused too much on typing into the computer. When asked about their anticipated response to the use of an AI scribe, 48% (n=904) were favorable, 33% (n=630) were neutral, and 19% (n=359) were unfavorable. Younger patients (ages 18-30 years) expressed more skepticism than those aged 51 years and older. Further, 42% (655/1567) of positive comments received indicated this technology could improve human interaction during their visits. Comments supported that the use of an AI scribe would enhance patient experience by allowing the clinician to focus on the patient. However, when asked about concerns regarding the AI scribe, 39% (515/1330) and 15% (203/1330) of comments expressed concerns about documentation accuracy and privacy, respectively. Providing previsit patient education and obtaining permission were viewed as very important.

**Conclusions:**

This patient survey showed that respondents are generally open to the use of an AI scribe program for EHR documentation to allow the clinician to focus on the patient during the actual encounter rather than the computer. Providing patient education and obtaining consent before using AI are important components to gain patient trust. Caution about the results is appropriate, given the low response rate and nonrepresentative profile.

## Introduction

The use of electronic health records (EHRs) has become ubiquitous in US medical practices [1]. The benefits of EHR for medical documentation include legibility, accuracy of record-keeping, enhanced ability to access laboratory data, placement of physician orders, interactive alerts, clinical pathways, plus timeliness and interoperability between health care institutions. Since the EHR is designed to be comprehensive, there are considerable and growing demands on clinicians for documentation of the myriad aspects of patient encounters. The time to complete EHR for clinic notes commonly exceeds the time associated with face-to-face visits between the patient and clinician, which may contribute to clinician burnout [2,3]. Research has documented that physicians can spend more than 15 hours per week solely on documentation [2].

Many clinical practices have used live human scribes in the last 10 years to address the substantial work required for medical documentation. Existing research finds that this support significantly lessens physician documentation burden and improves physician-patient communication [3]. With appropriate training, live scribes can effectively and efficiently transcribe and summarize the direct interactions between patients and clinicians, as well as integrate important information such as previous medical records, laboratory, and imaging tests [4]. The use of live scribes can markedly decrease the time and effort of the clinicians to document clinical notes, which may result in less time outside of clinic hours for documentation [5]. Since the use of live scribes is very common in current medical practice, patients are both accepting and supportive of their use [6]. However, there are significant limitations to scaling the usage of live scribes, including staff recruitment and cost.

Recently, there has been the emergence of digital scribes, which use generative artificial intelligence (AI) to transcribe human speech and then transform this into a standard progress note format [7]. There are other recent examples of the use of AI in health care delivery, with AI also entering the patient care landscape for patient navigation and medication adherence [8], and analyzing radiological imaging [9].

Use of AI digital transcriptions (also known as ambient voice technology, or “AI Scribes” may also help lessen the burden of clinical documentation, similar to the use of live scribes [10,11]. It may also enhance the patient experience during clinic visits compared to direct documentation by the provider, since the clinician may be able to spend more time interacting directly with the patient rather than documenting in the EHR during the visit. Initial reports about the use of AI Scribes show a strongly positive response from clinicians, since there is often decreased time and cognitive effort required for medical documentation [12-15]. Although most clinicians had a favorable view of their experience with the use of the AI scribe, the accuracy of the note is still problematic with regard to errors, length of the note, and note style requiring clinician effort to edit the note before finalization [11,12].

However, on a cautionary note, patients may be hesitant to accept use of an AI scribe due to a lack of familiarity or information, or preexisting beliefs about the process, accuracy, and integrity of the information shared between the patient and clinician. In anticipation of a planned deployment of an AI scribe program, our patient experience team surveyed our patients who receive primary care at the University of California, Davis ambulatory clinics. The intent of the survey was to understand the preexisting perceptions of our patients about the current state of EHR documentation and to anticipate both the positive and negative reactions that might be experienced before the rollout of an AI scribe program. In addition, this information could influence how we communicate about the AI scribe program, including the opportunity to opt out of participation.

## Methods

### Overview

We conducted this study from January 31, 2024, to February 12, 2024, at the University of California Davis Health (UCDH) in Sacramento, CA. UCDH is an academic medical center with 11 community medical group locations that provide care to a diverse catchment area of 6 million people across 33 Northern California counties.

A convenience sample of 9171 patients 18 years and older who had a UCDH primary care visit within the last year was recruited through the postclinic visit surveys via an email that was only sent in English. Patients spoke English as a primary language and were not incentivized to participate. Age, as well as self-identified demographic characteristics such as sex, race, and ethnicity, were taken from the electronic medical record (EMR). When available, these EMR data were collated with their email responses.

### Survey Design

A 17-question survey was designed by the UCDH Patient Experience Design team to capture patient perceptions and concerns about the anticipated future rollout of an AI scribe technology. The survey questions included a rating scale for some answers and open-ended responses for others.

The survey was developed and coded using Press Ganey’s Forsta Foundation tool, a JavaScript-based survey platform that assists in simplifying the programming of a survey. Once the survey was developed, it went through 5 functionality tests to ensure proper survey function and data collection.

The survey design took place over multiple iterations. With the goal of identifying possible sources of patient frustration or concern, survey design discussions focused on what insight would be needed to develop possible implementation approaches before the launch of an AI scribe technology within UC Davis Health.

A committee was established to provide feedback on survey design that consisted of 5 individuals: our Chief Medical Information Officer, a medical educator attending with patient research experience, a medical educator who leads digital health clinical programs for the health care system, a program manager responsible for patient experience survey design, and a qualitative survey design professional responsible, who facilitated the creation and execution of the survey. The survey was reviewed by the committee, which provided feedback on question inclusion, removal, and design. The full introduction to the survey and questions is included in Multimedia Appendix 1.

Participants received an email invitation with an option to opt out. Patients were provided the following brief description of the technology.

“To help take notes, UC Davis Health is testing a tool that looks similar to a cell phone. Upon being activated by the doctor, the tool listens to the conversation and automatically creates notes in the system*.*

As you participate in this survey, please focus on the note-taking capability and how you would like the use of this technology to work better for you. Please keep in mind that:

- The transcribed notes will be reviewed by the physician.
- Patients and families would be informed about the use of this device.”

Results were monitored hourly for the first 150 responses to ensure respondents were completing the survey and providing usable responses for open-ended questions. Since the study’s goal is to assist in the development of AI scribe deployment, respondents were required to answer all questions before proceeding. For questions seeking a numeric response (ie, 5-point scale), a score of 3 provided respondents the ability to offer a neutral response. Another batch of 150 was sent again to gauge their responses.

After the initial survey responses were reviewed, it was determined that the survey functioned well and did not require any modifications. However, the email request to the remaining patients (about 8800) was slightly modified, and the final version is contained in Multimedia Appendix 2, with the clarifying language involving the last paragraph noting that the “transcribed notes will be reviewed by the physician” and the “patients and families would be informed about the use of the device.”

The median length of time to complete the survey was 10 minutes and 33 seconds. The survey was provided via a secure and closed link, which allowed respondents to start the survey and complete it later. The shortest survey response was 2 minutes and 15 seconds. The longest was 256 hours and 25 minutes.

### Survey Analysis

The data were reported using descriptive summary statistics. Since this was a convenience sample survey, no statistical comparisons between groups were performed. Descriptive statistics were used to identify broad themes and to help guide the pilot implementation and deployment of the AI scribe program at UC Davis Health. Since the group of respondents may not have been representative of our clinic population, comparative statistical analysis of the respondent groups was deemed not justifiable. Incomplete responses were not used in the final analysis of this study.

An inductive coding framework was used for open-ended questions. The process entailed a review of all responses to a specific question and identifying response themes (which helped generate a code book). Each response was reviewed and assigned a descriptive label from the code book summarizing the basic theme of a response. A separate research team member evaluated the responses for alignment or discussion. Research team members met to ensure consensus on the application of codes, refine dimensions of existing codes, add new codes, develop categories, and identify theoretical direction. Disagreements among the team members were resolved through discussions to understand viewpoints, deliberate, and achieve consensus. An open-ended response could have multiple themes based on the patient’s input. Open-ended responses that were singular in nature were categorized as “other.” Statements made by patients that did not align with the subject matter were classified as “not applicable or NA” (ie, a question in the survey asks about a patient’s opinion regarding a provider taking notes, and the patient responds with negative statements about parking). Both members of the team are professionally trained survey designers.

### Ethical Considerations

This quality improvement study was reviewed by the UC Davis Health Institutional Review Board and deemed non–human subjects research, and therefore exempt from review, because the data came from patient surveys. Since this study was exempt from review and the participants were anonymous, we did not obtain informed consent, waivers, or privacy protections. Participants were not compensated for completing this survey.

## Results

The survey had an overall completion rate of about 21% (n=1893 respondents out of 9171 surveys). An additional 549 started the survey, but did not complete it. Partial responses were excluded from the survey results. All percentages calculated from 1893 respondents. The age, sex, race, and ethnicity data were extracted from the EMR and collated for each patient response. The respondents’ demographics were predominantly female (1205/1893, 64%), ages 51 years and older (1649/1893, 87.1%), and White (1312/1893, 69%; see Table 1)*.* As noted in the Methods section, patients self-identified their race and ethnicity. We compared these percentages to our patient population at UC Davis Health, using our Epic EHR, and accessed the active clinic patient registry for any interactions in the past 2 years. The demographic profile of this group was as follows: female 53.7% (207,947/387,512; total clinic population), ages 50 years and older 44.5% (172,500/387,512), and White (not Hispanic) 37.3% (144,836/387,512).

**Table 1:** Survey responses and respondent demographics.

Responses and respondent demographics^a^				Results
**Surveys**				
	Total sent, n		9171	
	Responses, n (%)		1893 (20.6)	
**Demographic variables (N=1893), n (%)**				
	**Sex**			
		Female	1205 (63.7)	
		Male	688 (36.3)	
	**Age (years)**			
		18-30	64 (3.4)	
		31-40	60 (3.2)	
		41-50	120 (6.3)	
		51-60	285 (15.1)	
		61-70	622 (32.9)	
		71+	742 (39.2)	
	**Race and ethnicity**			
		White or Caucasian	1312 (69.3)	
		Multirace (Ethnicity)	154 (8.1)	
		Latinx or Hispanic	130 (6.9)	
		Unknown	105 (5.5)	
		Asian	86 (4.5)	
		Other^b^	57 (3)	
		Black or African American	42 (2.2)	
		Native Hawaiian or Other Pacific Islander	5 (0.3)	
		American Indian or Alaska Native	2 (0.1)	

^a^Before introducing an AI (artificial intelligence) Scribe program, UC Davis Health primary care patients were sent an email questionnaire about their perceptions to determine how the program should be implemented.

^b^American Indian or Alaskan Native, Native Hawaiian, or other Pacific Islander were combined in the “Other” category for reporting, since those numbers were small.

The survey was performed as a quality improvement project, rather than a scientific investigation. Given the potentially nonrepresentative patient sample, we did not perform comparative statistical analysis between the groups. Thus, these results should be considered as indicating broad informal trends.

Patients’ perceptions of current clinician notetaking practices were generally positive, with 71% (1349/1893 responses) feeling very heard or somewhat heard and 79% (1490/1893 responses) indicating their doctors were engaging in their health discussion while taking notes during their primary care provider (PCP) appointment. However, younger patients (ages 40 years and younger) and Asian and Latinx or Hispanic patients expressed more critical views, citing a lack of engagement and limited interest from physicians during notetaking. A review of actual open-ended patient comments revealed that 62 responses were not applicable to the question, leaving 1831 responses. The comments related to clinician EMR documentation demonstrated that 29% (530/1831 responses) were very positive and the patients felt “heard” by their provider, and another 24% (439/1831) reported that the notetaking did not bother them, since this was a normal routine. 21% (385/1831) reported that it was a “necessary evil” because documentation is required. 23% (416/1831) reported that when the provider took notes into the computer during the visit, it felt very impersonal to them, that the physician was distracted, and sometimes they felt disrespected. An example of a typical positive comment was “I feel she is listening and accurately recording my info since she is recording as we speak.” Examples of negative comments include: “I feel the need to slow down and even stop talking until my PCP completes her typing,” “Feels a bit impersonal, like he’s just entering data to tick boxes, etc. rather than really discuss the issue with me,” and “I realize it’s a necessity – but would rather they just be able to listen.”

When asked about how the AI scribe technology would impact the overall clinic visit, 48% (904/1893 responses) of patients responded positively, 33% (630/1893) were neutral, and 19% (359/1893) felt it would have a negative impact on the patient-physician interaction.

Overall, regardless of age, more patients had a favorable perception about the use of AI scribes than neutral or negative (see Figure S1 in Multimedia Appendix 3)*.* Looking at the trends in perceptions about AI scribe technology suggested some differences by age. Younger patients (aged 18-30 y) appeared to have a higher frequency of concerns about the potential negative impact (31%, 20/64 responses) compared to older groups. Interestingly, patients aged 50 years and older seemed to have the most favorable perceptions and the least skepticism (18%, 300/1649 responses).

Figure S2 in Multimedia Appendix 3 shows the breakdown of perceptions by race and ethnicity, which varied among the groups. The majority of patients had positive perceptions across all groups, ranging from 46% (598/1312) “White” responses to 67% (58/86) “Asian” responses. Patients who identified as “Asian” or “Black” seemed to have more positive perceptions about the use of a voice technology and lower rates of skepticism. Negative perceptions were around 20% (323/1660) for the remaining groups; 20% (257/1312) “White” responses were negative, 19% (30/154) “Multirace (Ethnicity)” responses were negative, 18% (23/130) “Latinx or Hispanic” responses, and 20% (13/64) “Other” responses were negative.

The open-ended comments about the perceived benefits were collated and grouped by themes. Table 2 shows these themes in descending order. The respondents listed the following anticipated benefits for an AI scribe: improve human-to-human interactions (41.8%, 655/1567 comments), accuracy of notes (28%, 435/1567), benefits to doctor and staff (15%, 231/1567), generally positive (6%, 98/1567), and transparency (4%, 58/1567). The listing of potential benefits varied by age, with 57% (28/49 responses) of patients aged between 31 and 40 years listing “improve human elements” as the most frequent. There was less variation when comparing the results by race and ethnicity. Positive open-ended comments about the “improve human elements” included: “It will free up the doctor to read emotion and other non-verbal cues and ask follow-up questions,” and “If it allows the doctors to see more patients, it could decrease the time it takes to get an appointment.” Positive comments about “accuracy of notes” include: “Better information regarding my complaints and symptoms. Nothing missed,” and “It will more accurately reflect our conversation. I often read the notes in the After Visit Summary and find errors….”

Numerous concerns about the AI scribe program were noted by the survey respondents (see Table 3)*.* The most commonly mentioned were related to note accuracy (39%, 515/1330 comments), privacy and security (15%, 203/1330), negative feelings about being recorded (13%, 177/1330), and general feelings that it was bad for the physician and staff (10%, 139/1330), plus others. These concerns were common among those respondents who thought that the digital technology would have a negative impact. Younger respondents expressed the greatest skepticism over note accuracy, privacy, and security. Key comments focused on the risks that the recordings might be discovered by hackers, which would be a violation of privacy. There were both positive and negative comments about the potential accuracy of the digital transcriptions. Some felt that the transcribed clinic notes would be more accurate, while others were worried that there was a higher risk of containing inaccurate statements (transcription errors). Since the survey was done preimplementation, this probably reflects the respondents’ best speculations about the effectiveness of the AI scribe technology.

Patients were queried about the best time to be informed about the AI scribe program. It was clear that patients preferred early notification, either when the appointment is made (43%, 809/1893 responses), upon arrival at the examination room (32%, 602/1893), or upon arrival at the clinic (15%, 276/1893). When given choices about how they would be informed, patients preferred verbal discussion (57%, 1085/1893 responses), followed by email (45%, 854/1893), text (28%, 532/1893), and then via brochure (24%, 460/1893), followed by miscellaneous. Patients had fewer clear preferences about who might provide the notifications, with a slight preference to hear from the physician or resident over nursing or support staff. When asked about the preferred method to be informed about the use of AI scribe technology before the clinic visit, the respondents most preferred an email (63%, 130/206 respondents), text message (40%, 82/206 respondents), or verbal discussion (38%, 78/206 respondents).

**Table 2:** Thematic benefits of artificial intelligence (AI) scribe.

Response themes^a^	Coded comments, n (%)
Improve human elements	655 (41.8)
Accuracy of notes	435 (27.8)
Beneficial to the doctor and staff	231 (14.7)
General positive	98 (6.3)
Transparency	58 (3.7)
Mixed	36 (2.3)
Improve access	28 (1.8)
Other	26 (1.7)

^a^Patients responded to open-ended questions. These comments were collated and grouped by themes, with the frequency of themes listed in descending order.

**Table 3:** Thematic concerns of artificial intelligence (AI) scribe.

Response themes^a^	Coded comments, n (%)
Accuracy of notes	515 (38.7)
Privacy and security	203 (15.3)
Problem with being recorded	177 (13.3)
Bad for the physician and staff	139 (10.5)
Human element	126 (9.5)
Other (technical failure, billing, incorrect *ICD* code, referrals, etc)	58 (4.4)
Transparency (MyChart, Notes summary, etc.)	22 (1.7)
General negative	90 (6.8)

^a^Patients responded to open-ended questions about their concerns related to the implantation of an AI scribe program. These comments were collated and grouped by theme, with the frequency of each theme listed in descending order.

## Discussion

### Principal Findings

This survey of UCDH primary care patients in anticipation of a deployment of our AI scribe program helped inform our team about the perceived benefits and concerns to shape our implementation. This convenience sample of nearly 1900 patients, with a response rate of about 20% (1893/9171), was sufficient to influence the implementation process. We believe that these results can support other health care institutions to implement patient-centered, culturally competent AI scribe programs.

It is important to note upfront that the demographic profile of the patients who responded to the survey was less representative of the overall outpatient clinic population at UC Davis Health, skewing more strongly to those who were female, were ages 50 years and older, and White compared to our overall clinic population. Thus, interpretation of the survey results should be tempered with this knowledge. Nonetheless, we believe there is value in reporting these results, given the relatively large number of responses and broad trends.

There were several overall trends expressed by the respondents that were supportive of the AI scribe program, as well as cautionary notes. Of the survey respondents (predeployment of an AI scribe program), most patients felt that their physicians were currently attentive during the visit (71% 1349/1893), and patients accepted that EHR documentation was needed to ensure an accurate record of patient encounters. Nonetheless, 22% (416/1893) of respondents were put off when their providers were typing into the computer during the visit, feeling that the clinician was too focused on the documentation and paying less direct attention to the patient. This may reflect different interaction styles between physicians and their patients, rather than a rejection that physicians perform documentation during the clinic visit. Most patients appear to have realistic views about how clinicians must balance their approach to directly interacting and listening to them with the burdens of documentation. Nonetheless, if using an AI scribe during the visit frees the physician to directly interact with the patient, this is likely to be perceived positively.

In support of this interpretation, a plurality of respondents viewed the use of an AI scribe in a positive light (42%, 655/1567 positive open-ended comments), noting that this might enhance the clinician’s ability to focus on the patient and less on documentation during the encounter. Other positive benefits included improved note accuracy, reduced workload for the clinician and staff, and improved transparency. Although we are cautious not to overinterpret the differences based on age and race and ethnicity (since the groups were not subjected to formal statistical comparisons), there were interesting trends. For example, in contrast to our expectations, patients aged 50 years and older appeared to be more positive about the potential benefits of use of an AI scribe compared to a younger cohort. The younger patients expressed more concerns about privacy and security with regard to being recorded as part of the AI scribe technology, as well as accuracy. It appeared that non-White patients expressed positive impressions about the use of the AI scribe at least as often as White patients.

Nonetheless, although there were negative comments or concerns expressed, only 19% (359/1893) of respondents thought that the overall impact would be negative, whereas 48% (904/1893) thought that it would be positive. Thus, this may reflect anticipatory anxiety, and this could be readily addressed by both previsit patient education and actual experience.

### Comparison to Previous Work

There are currently limited studies that report patient experiences with AI scribe technology, and the responses have been generally favorable. Most research to date has focused on time saved to physicians or on using AI to generate medical advice, while very little has been done to measure patient attitudes about AI scribes during clinic encounters. Cao et al [7] reported their experience with the use of the Dragon Ambient eXperience (DAX; Nuance & Microsoft) system in a dermatology practice. In a limited survey of patients (score 1-5, 5 being “strongly agree”), there was high favorability on the following questions: “The provider spent less time typing on their computer” (mean score 4.5), “My visit felt more like a personable conversation” (mean score 4.4), and “The provider seemed more focused on me during the visit” (mean score 3.9). A group of Northern California Kaiser Permanente physicians and researchers published their initial experience with an AI scribe program [16]. The primary focus of this paper was on the implementation of the software-based medical dictation technology from a provider standpoint (accuracy of note, usability, and effectiveness). They described their patient-facing educational materials as part of their permission process that included a verbal summary of the AI scribe to the patients, as well as laminated posters. Between October and December 2023, the AI scribe was rapidly implemented, with over 3400 physician users and more than 300,000 patient encounters. In a limited survey of 21 patients from a single clinic site, 71% of respondents noted that they spent more time talking with their physicians, and 81% reported that physicians spent less time looking at their computers during the encounter. The patient responses to early experience with the AI scribe match up well with our patient survey results. In a news article about the Kaiser Permanente study, Feldheim [17] noted that the benefits of the AI scribe extended beyond the time savings for the providers: “they may help restore the fundamental human connection at the heart of medicine.”

It is worth considering potential explanations about why there appears to be a difference in attitude between older and younger respondents, even though our survey was not designed to explore these differences in detail. Several papers appear to offer clues about what might be underlying these differential attitudes. Older adults, especially those who are comfortable with technology, are willing to share their personal information with family and hospitals (compared to government agencies) [18]. Older adults are selective about what information they share, but if they perceive that these services are used to maintain and promote health, then they are willing to share that information, even if they have concerns about privacy [18]. Kauttonen et al [19] conducted a web-based survey of Finnish participants to gauge trust and acceptance of artificial intelligence when adapted into health care delivery. They found that age was a factor in the acceptance of the use of AI in health care, but not always a consistent trend. Instead, the combination of familiarity with technology along with age appeared to be more important. For example, those who were less familiar with AI were the least trustful. Interestingly, though, people aged 70 years and older were the most positive about AI when balanced with trade-offs. There were significant confounders based on the type of AI scenario presented to the participant. In a study of German patients at a tertiary care center, more than 50% of participants had positive attitudes about the use of AI in health care, and only about 5% had serious reservations. In contrast to our study results, older patients and women expressed more caution about AI in health care [20]. The key to acceptance is that the patients insist that the use of AI be under the control of their physician.

Younger patients may have a greater understanding of technology, particularly AI. In a survey report offered by Cisco, they found that about 48% of respondents agreed that AI could be useful to improve their lives. However, there were serious concerns, especially about the use of generative AI, even though few were very familiar with this technology. In particular, there was substantial concern about privacy protections, with the youngest consumers (42% of those aged between 18 and 34 y) actively engaged in protecting their privacy, and this was 7 times higher than those aged 75 years and older [21]. A small group of young adults (25-34 years) was provided with a focus group survey about AI-driven mobile health technology [22]. Many expressed enthusiasm (and skepticism) about the emerging technology. Prominent among the perceived risks were concerns about data privacy and the need for human supervision.

These studies seem to highlight several themes relevant to the use of an AI scribe. One is that there is limited familiarity with the new technology. The degree of enthusiasm seems closely aligned with both familiarity with technology and the perception of whether it can enhance health care delivery for the patient. It may be that older patients are more accepting that the AI scribe can unburden the physician’s responsibility for clinic documentation, whereas younger patients have greater concerns about privacy and lack of control over their personal information when their data is fed into a generative AI program. As noted in the Conclusions section, education about the process and protections for privacy are key to acceptance and trust.

### Strengths and Limitations

There are several strengths to this study. First, is that we received a relatively large number of responses to the survey, representing about 20% (1893/9171) of those who were contacted. Second, we queried about both the current patient-physician interactions as well as anticipating patient responses to the eventual implementation of an AI scribe voice technology for medical documentation. Third, we obtained actionable information that helped modify the future rollout of the program. For example, we prepared our users with information about the system’s security and data retention policies so they would be prepared to respond to expected patient concerns. Finally, we identified that patient education (in various manners) would be a critical element for patient acceptance of the AI scribe.

Several important limitations affect the interpretation of the survey results. A primary limitation is that the demographic profile of those who responded was not representative of the clinic population, as determined by the self-identification of the patients noted in our EMR. Since this was a convenience sample and not expected to be fully representative of our patient population, this limits our ability to precisely compare the differing responses among the varying demographics and perform statistical analysis. The survey was only sent to English-speaking patients and was sent by email, so the perspectives of non-English speaking patients and those with limited digital access or literacy on this technology are not known. Given this limitation, the best we could expect was to understand broad themes. It is worth acknowledging that the demographic characteristics of the respondents are probably more representative of who chose to respond to the survey rather than the overall population of patients who receive their health care at our institution. Since survey respondents skewed older and white, we are uncertain whether a younger, more diverse population would actually experience this technology differently. The response rate was only 20%, so this also limits the generalizability of the results. Thus, it will be important to monitor patient reactions to the rollout of the AI scribe over time, since the actual patient experience may vary from the anticipated one. To that point, we note that this survey had a high nonresponse rate. Finally, we intentionally shared information about the technology with participants that was simple and written for a lower literacy competency. While we believed this was appropriate, some participants may have wished for a deeper explanation of the technology involved.

Nonetheless, the survey provided valuable guidance on how to communicate about the use of AI scribe and gain patient consent for a new technology that some may find uncomfortable or threatening. A recent study by Lawrence et al [23] describes a quality improvement project focusing on determining the best approaches to obtaining patent consent before using the AI scribe during a clinic visit. The keys to successfully obtaining patient consent included establishing patient-physician trust, targeted education (more than a single modality and a flexible approach to answering patient questions), and honest discussions about potential vulnerabilities (risk of security breaches, inaccurate documentation, and risk of medical errors). When basic information about the AI scribe was provided to the patients, 81.6% consented. This study supports the findings that we obtained from our own patient survey, that most patients would accept the AI scribe, if introduced with appropriate education and permission sought beforehand.

### Conclusions and Recommendations

We learned that although many patients understand and accept that providers commonly complete their medical documentation during the actual clinic visit to increase efficiency, a significant proportion feel that this interferes with effective and empathetic communication. Using AI Scribe technology may allow clinicians to spend more time in direct dialogue with patients rather than typing into the EHR and staring at a computer screen. Key to the acceptance of an AI Scribe program is to educate the patient about the program in advance (through placard notifications in the clinic, EMR notices, or verbal discussions) and obtain explicit verbal permission from the patient before use. The patient must be allowed the option to opt out. Verbal consent should be documented in the patient’s clinic note.

## Appendix

Multimedia Appendix 1 Questionnaire introduction and questions.

Multimedia Appendix 2 Email introduction to patient survey before deployment of the artificial intelligence (AI) scribe, plus discussion about educating patients about the AI scribe technology.

Multimedia Appendix 3 Primary care patient perceptions about an artificial intelligence (AI) scribe program by race and age.

## Abbreviations

AI: artificial intelligence
EHR: electronic health record
EMR: electronic medical record
PCP: primary care provider
UCDH: University of California, Davis Health

## Data Availability

The datasets generated or analyzed during this study are available from the corresponding author on reasonable request.
