# Supplementary material for: Patient Attitudes Toward Ambient Voice Technology: Preimplementation Patient Survey in an Academic Medical Center
Source: JMIR Med Inform. 2025 Nov 27;13:e77901. doi: 10.2196/77901 (PMC12699246; doi:10.2196/77901)
Supplement: Multimedia Appendix 1 [file medinform_v13i1e77901_app1.docx]

***Appendix 1***

*Questionnaire introduction and questions.*

UC Davis Health is going to start testing new software to help physicians take notes during care visits. Today you will often find physicians, residents, or other support staff taking notes on a computer while you speak. It is not uncommon to see physicians taking notes directly into the computer as part of your health record. Notes are a very important part of health care as they allow communication between members of your care team, help assure that the details of your condition are available for decision-making, and support quality improvement efforts as well as billing.

We are looking to bring new tools to improve that experience and allow for physicians to shift their focus to you, by using technology that listens to the conversation and creates notes automatically. The device is a typical smartphone in the room with the microphone on. Patients and families would be informed that it is present and will be active during their doctor’s visit. The visit would be very similar to today, but the provider is less likely to be taking notes into a computer. They may still use the computer to enter orders or instructions for after your visit.

We would like to learn your opinion about this type of tool being used in a care environment as we start to pilot the new technology. Understanding your thoughts and concerns will help us develop the correct approach to this type of technology.

1. Do you have a primary care physician/doctor at UCDH today?
   1. Yes
   2. No (terminate)
   3. I am not sure (terminate)
2. How long ago did you last see your primary care physician? (will this only cascade for ‘a’ above.?)
   1. Within the last 3 months?
   2. 4 to 6 months?
   3. 7 to 9 months?
   4. 10 to 12 months?
   5. Over 12 months ago?
3. When thinking back to a recent visit with your primary care physician, about what percentage of the time is the doctor looking at you and engaging in discussion or evaluation versus looking/taking notes or typing information into a computer? Use your best guess, we know it won’t be 100% accurate.
   1. They spent 100% of the time looking at and evaluating me
   2. They spent 90% of the time looking at and evaluating me
   3. They spent 80% of the time looking at and evaluating me
   4. They spent 70% of the time looking at and evaluating me
   5. They spent 60% of the time looking at and evaluating me
   6. They spent 50% of the time looking at and evaluating me
   7. They spent 40% of the time looking at and evaluating me
   8. They spent 30% of the time looking at and evaluating me
   9. They spent 20% or less of the time looking at and evaluating me
4. How do you feel today when you see the physician talking with you but either focusing on taking notes or putting information directly into a computer? What emotions do you feel?
5. When doctors are taking notes or entering in information into a computer, how much do you feel listened to on a 5-point scale?
6. When doctors are taking notes or entering in information into a computer, how much do you feel the doctor is taking an interest in understanding your health issues to make the best decisions for you on a 5-point scale? A key element to this new technology is the use of a recording device that listens to the conversation and transcribes the discussion into notes. You will always be made aware that the devices are present and when they are active.
7. What benefits do you feel this technology will provide to you as a patient?
8. When you think about the approach described above, what concerns do you have?
9. What is your single largest concern? (cascade if not none?)
   1. What should UCDH do to help address your top concern?
10. How concerned are you about privacy of your health information with this technology on a 3-point scale?
11. How concerned are you about accuracy of the notes that is created on a 3-point scale?
12. How will using this type of technology impact your perceptions of your UCDH primary care visit?
    1. It will improve my perceptions of my PCP visit with UCDH
    2. It will decrease my perceptions of my PCP visit with UCDH
    3. It will not impact my PCP experience at all
13. When should patients be told about the presence of this technology?
    1. When their appointment is made
    2. Just prior to their appointment day – maybe a day or two prior to visit
    3. Upon their arrival at the clinic
    4. Upon their arrival to their exam room
14. Why did you provide that answer?
15. How should we tell patients about the technology at a summary level?
    1. Text message
    2. Email
    3. Brochure in the clinic itself
    4. A brochure mailed to the patient prior to their visit
    5. Verbal discussion (if so, by whom? A video? Docs may not know details of the behind-the scenes)
16. IF 15.5: Who should be the one to provide that verbal discussion the technology?
17. The physician or resident
18. Nurse staff
19. Other support staff, such as the front desk staff
20. What other thoughts do you have about this topic?
