# Supplementary material for: Patient Attitudes Toward Ambient Voice Technology: Preimplementation Patient Survey in an Academic Medical Center
Source: JMIR Med Inform. 2025 Nov 27;13:e77901. doi: 10.2196/77901 (PMC12699246; doi:10.2196/77901)
Supplement: Multimedia Appendix 2 [file medinform_v13i1e77901_app2.docx]

**Appendix 2**

Respondents were not provided significant detail about AI scribe technology, the platforms being considered for AI scribe, or on how AI scribe/transcription software works. The objective was to capture how patients would feel about the topic similarly to how it might be introduced in a healthcare setting – with limited explanation or detail. Capturing a respondent’s overall understanding of AI scribe technology was considered but ultimately ruled out due to concerns that asking questions about awareness could inadvertently influence responses and gauging respondent knowledge was less important than potentially influencing respondent viewpoints or feelings about the topic. To minimize influencing opinions and provide all patients a basic understanding of the topic and technology, the following email introduction was provided to help inform patients on the topic:

Current setting: During a meeting with your primary care doctor, you will see physicians, residents, or other support staff taking notes on a computer while you speak. They are capturing things such as symptoms, concerns and other details of your visit. These notes are very important as they allow for communication between members of your care team, help assure that the details of your condition are available for decision-making, support quality improvement efforts, and even assist with things such as billing.

Future setting: To help take notes, UC Davis Health is testing a tool that looks like a cell phone. Upon being activated by the doctor, the tool listens to the conversation and automatically creates notes in the system.

As you participate in this survey, please focus on the notes taking capability and how you would like the use of this technology to work better for you. Please keep in mind that

- The transcribed notes will be reviewed by the physician.

- Patients and families would be informed about the use of this device.
