# Supplementary material for: Patient Attitudes Toward Ambient Voice Technology: Preimplementation Patient Survey in an Academic Medical Center
Source: JMIR Med Inform. 2025 Nov 27;13:e77901. doi: 10.2196/77901 (PMC12699246; doi:10.2196/77901)

**Figure S1.** Primary care patient perceptions about an AI scribe program by age. Responses from patients about their perception of an AI scribe program, stratified by age. AI: artificial intelligence.


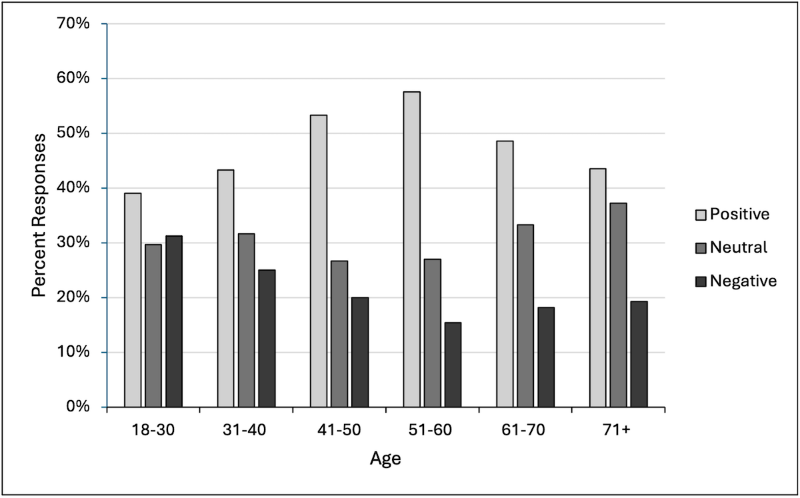


**Figure S2.** Primary care patient perceptions about an AI scribe program by race. Responses from patients about their perception of an AI scribe program, stratified by race and ethnicity. AI: artificial intelligence.


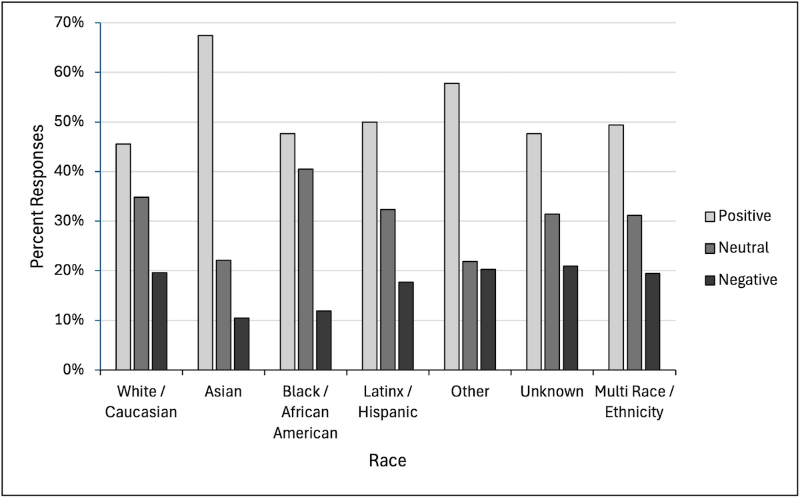

Supplement: Multimedia Appendix 3 [file medinform_v13i1e77901_app3.docx]
